# Remote Driven and Read MEMS Sensors for Harsh Environments

**DOI:** 10.3390/s131014175

**Published:** 2013-10-21

**Authors:** Aaron J. Knobloch, Faisal R. Ahmad, Dan W. Sexton, David W. Vernooy

**Affiliations:** 1 General Electric Global Research, One Research Circle, Niskayuna, NY 12309, USA; E-Mails: ahmad@ge.com (F.R.A.); sextonda@ge.com (D.W.S.); 2 General Electric Power & Water, One River Road, Schenectady, NY 12345, USA; E-Mail: vernooy@ge.com

**Keywords:** acoustic resonator, electromagnetic coupling, high temperature, microelectromechanical devices

## Abstract

The utilization of high accuracy sensors in harsh environments has been limited by the temperature constraints of the control electronics that must be co-located with the sensor. Several methods of remote interrogation for resonant sensors are presented in this paper which would allow these sensors to be extended to harsh environments. This work in particular demonstrates for the first time the ability to acoustically drive a silicon comb drive resonator into resonance and electromagnetically couple to the resonator to read its frequency. The performance of this system was studied as a function of standoff distance demonstrating the ability to excite and read the device from 22 cm when limited to drive powers of 30 mW. A feedback architecture was implemented that allowed the resonator to be driven into resonance from broadband noise and a standoff distance of 15 cm was demonstrated. It is emphasized that no junction-based electronic device was required to be co-located with the resonator, opening the door for the use of silicon-based, high accuracy MEMS devices in high temperature wireless applications.

## 1. Introduction

There is a need for high accuracy, repeatable and reproducible sensors that can operate in harsh or constrained environments. In these situations, temperature limitations, physical access or other factors rule out the possibility of using batteries or other “active” energy harvesting techniques to power the sensors and new sensor architectures and interrogating techniques must be developed.

Remote interrogation of sensors is accomplished in productized sensors today in one of three ways. In the first method, a battery is used at the sensor node which enables the use of a local microprocessor for signal conditioning and communications uplink. There are challenges incorporating this technology in applications where size, weight, power, cost and lifetime at elevated temperature of the sensor are important. In a second method, energy is harvested to locally power the sensor node [1–4]. This class of sensors can accomplish battery-free operation, but will require either a rectifying technology (*i.e.*, semiconductor junction) [5] or other robust energy harvesting technique to allow the sensor node to function. This will introduce temperature limitations in the applications that the sensor can be used in.

A third method is to eliminate the need for a direct energy harvesting technology. An example of this method is surface acoustic wave (SAW) technology [6,7] and more recently, lamb wave resonators in AlN [8]. Gratings written into these devices can serve as RF time delays so that energy is temporarily stored and then reflected back to the interrogator. This enables time discrimination against diffuse backscatter. Furthermore, the delay can be made sensitive to temperature, pressure and strain. Another example of this class of sensor is a simple resonant circuit such as an LC resonator [9–12] at intermediate frequencies or transmission line resonator at high frequencies. Proper geometrical design can make either the inductor or capacitor (or both) sensitive to the variable of interest and changes in the resonant frequency (or sensor impedance) can be read out using interrogators similar to “grid-dip” meters or RF reflectometers familiar in radio technology. Yet, there are few examples where this approach has yielded robust results with high accuracy in harsh environments.

In order to address this challenge, high Q silicon-based MEMS micromechanical resonators are considered [13–16]. These devices are considered “world-class” when applied, for example, to the pressure sensing market and have been in mass production since the early 1990s. They maintain the inherent mechanical robustness of silicon while leveraging wafer-scale processing for high volume and low cost. Another very important benefit of these devices is that they convert the measured variable into a frequency output [17,18]. With proper design, the fact that the oscillation frequency of the MEMS resonator becomes proportional to the measurand of interest can be a distinct advantage. In particular, the output of the device can now be considered digital (*i.e.*, a frequency output that can be “counted”) so that as long as the signal can be detected, temperature induced amplitude variations do not affect the accuracy of the sensor. In current productized formats, these devices are used in an electrically interrogated, closed-loop topology thereby limiting their application in harsh environments due to their use of piezoresistors to detect the resonant oscillations of the device.

A combination of a high Q resonator with the ability to remotely readout the sensor without local, active electronics could allow these devices to be used up to temperatures at which silicon creep becomes an issue (typically > 350 °C and < 500 °C). However, it is necessary to develop a mechanism to deliver energy to the mechanical structure at the right frequency to drive the oscillations, and then a second mechanism is required to read out that frequency. Key factors considered were to try to minimize power consumption and complexity of the interrogator, while maximizing the standoff distance or “read range”.

One method to excite and read a MEMS resonator remotely would be through optically modulated signal and reading a Fabry Perot cavity formed by the resonator and the vacuum cavity around the sensor. The resonator oscillates due to the heat absorbed by heavily doped silicon at the wavelength of the modulation signal. This technique was demonstrated in the context of high accuracy pressure measurements needed to monitor a geothermal well [19,20]. An optimized resonator for this application was packaged as part of a fiber optical cable and deployed in a geothermal well at over 1200 m below ground seeing temperatures in excess of 200 °C [21]. In this approach, the interrogator was located at the top of the well.

One drawback of the optical approach is the size and integration of the sensor into an optical cable along with the size and complexity of the interrogator. Alternatively, we have demonstrated that excitation energy can be delivered wirelessly to the resonator (in this case a comb drive) through induction such that it oscillates at its resonant frequency. This frequency was then wirelessly, inductively read through a separate LC resonator formed from a different set of comb fingers at a separate frequency [22].

In contrast, this work demonstrates a new method where the comb drive is driven into resonance wirelessly through the use of acoustics and read wirelessly through inductive coupling. The method described here demonstrates more than a 2× improvement in standoff distance over the prior work and a closed loop feedback system allowing the resonator to be driven from broadband noise.

**Figure 1.**
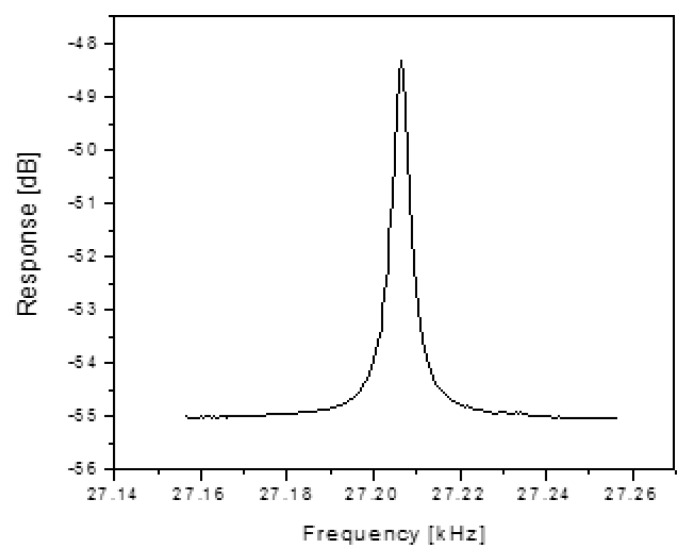
Comb drive resonators used for these experiments had a center frequency of 27.2 kHz, linewidth of 4 Hz, and therefore, a Q of 6800 (reproduced from [22] with permission).

Silicon comb drive resonators used for this work were based on existing GE production devices with a dual set of comb teeth with a gap size of 5 μm and an overall length of 500 μm. Details of the device are similar to that in [14,23]. Wafer bonding is used to isolate these devices in vacuum such that they have a characteristic mechanical resonant frequency of approximately 30 kHz and a Q of upwards of 6500. The top wafer of the structure has a diaphragm that is physically connected to the comb drive in such a way that the deflection of the diaphragm modulates the resonant frequency of the comb drive. Measurement of the resonant frequency and Q for these comb drives was made using a network analyzer to sweep the frequency and measure the output. Figure 1 shows the frequency response of a typical resonator. This characterization is important in order to gage the performance of the wireless methods with respect to required drive power, detected signal levels, signal distortion, resonant frequency and loop gain (or loss).

## 2. Acoustic Drive

**Figure 2.**
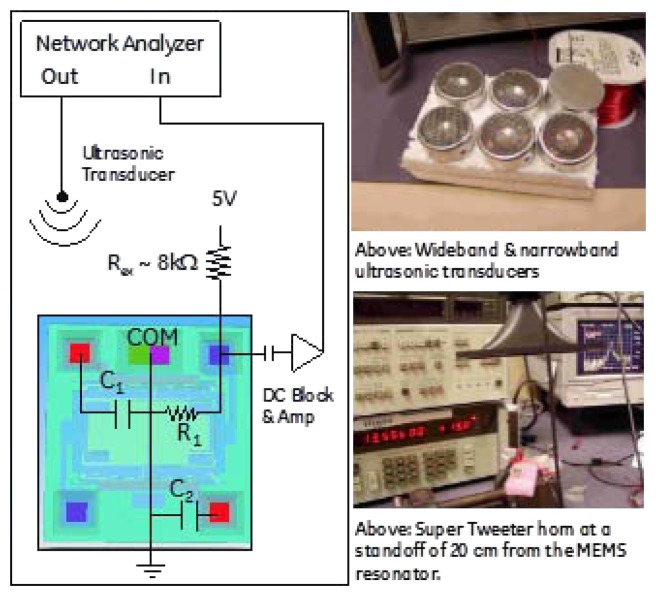
Schematic of the setup used to drive the comb drive at its resonance of 27.224 kHz. The picture on the left shows a top view of the die with electrical representation of the comb drive. The right images show examples of the acoustic transducers used to drive the resonator.

**Figure 3.**
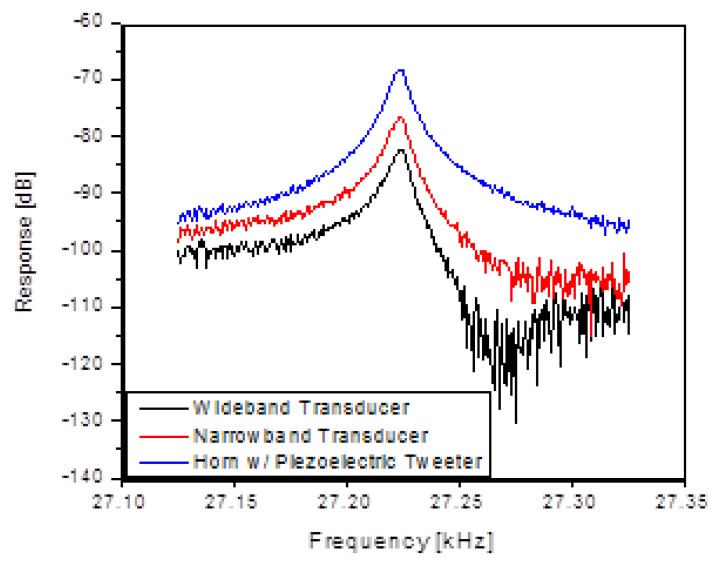
Demonstration of the ability of each of the transducers to drive the MEMS comb drive into resonance as measured by the piezoresistors on the die.

The first architecture considered was acoustically driving the silicon comb drive into resonance and measuring the frequency response using the piezoresistors located as part of the comb drive. In this case, the electrical aspects of the MEMS device were not important for the drive-side response. Figure 2 shows the experimental setup and transducers used to measure the efficiency of acoustic drive. Several ultrasonic driving techniques were tried with success, and in the end a low cost COTS quasi-resonant ultrasonic transducer (TR89/B, Type 31, Massa Products Corporation, Hingham, MA, USA) produced the most reliable results. With impedance matching, it is possible to turn this into a very narrowband transducer. However, it was used un-matched to allow for varying resonant frequencies in the device and driven directly from a 50 Ω source. Figure 3 shows response of the MEMS resonator as a function of the acoustic transducer type.

The Massa device is specified for a transmitting sensitivity of 25 dB compared to 1 μbar/V at 1 foot. When the acoustic impedance, Z_a_, is known, the sound pressure, p, can be related to the sound intensity, I, in power per unit area as:

(1) $$p=\sqrt{Z_{a}I}=\sqrt{\frac{Z_{a}P_{\text{drive}}}{A}}\propto \frac{1}{r}$$

where P_drive_ is acoustic drive power, A is area and r is the standoff distance. This relationship shows that the pressure (proportionally, the force on the die diaphragm) should fall off linearly with distance between the MEMS die and the transducer, and should increase linearly with the drive voltage, or, equivalently, as the square root of the sound power delivered by the transducer. In a driven oscillator, the displacement amplitude will be proportional to the driving force amplitude, so that the induced capacitance change will be proportional to pressure. Hence, it is expected that the power level of the detected resonance signal will fall off as the square of the distance between the transducer and the sensor and will be proportional to the drive power to the transducer.

**Figure 4.**
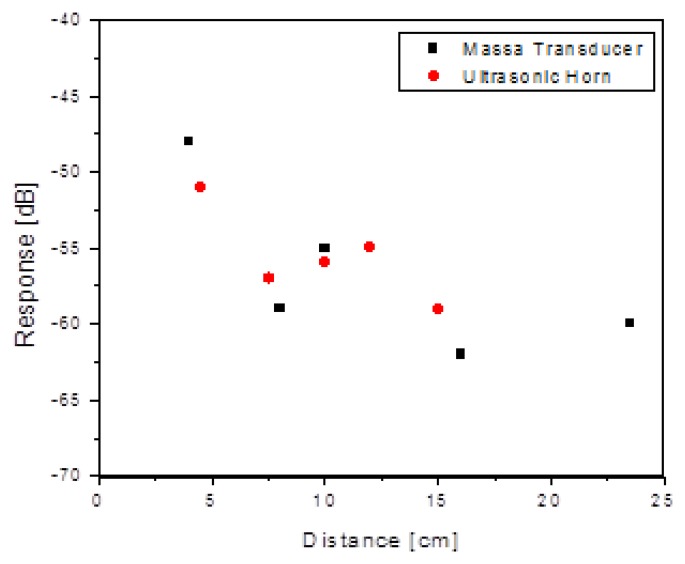
Signal response levels as a function of standoff distance for acoustic drive and wired (piezoresistive) read.

As demonstrated in Figure 4, the expected 6 dB/octave dependence on acoustic power was verified, as was the dependence on acoustic drive level (data is shown later in Figure 10). Finally, within a range of about ±10 cm, there was not a significant dependence on lateral mismatch between acoustic transducer and the sensor. Overall, the success of the acoustic driving was somewhat surprising because the only way to mechanically couple into the resonator is via the diaphragm. The mechanical resonance frequency of the diaphragm (nominally 100 kHz) is an important factor, but something that was not examined in detail. As a final comment, it is likely that more advanced acoustic design techniques (such as phased array transducers) could be used to optimize the excitation distance, power delivery and impedance matching to the die. These techniques are common in ultrasound technology but were not employed here.

## 3. Inductive Read

Inspired by the capacitive electrical equivalent of the comb drive, a readout method based on induction was developed. This capacitance can be resonated with an inductance to form an LC “tank” circuit. Energizing this tank circuit with an external drive coil at its electrical resonance frequency:

(2) $$\omega_{s}=\frac{1}{\sqrt{LC}}$$

should produce a voltage at the sensor. If the sensor is already driven into oscillation (for example, acoustically), the comb drive capacitor will have oscillating voltages at both the mechanical drive frequency, ω_m_ and the electrical drive frequency, ω_e_. The square law in the force-voltage relationship of a comb drive with capacitor C_r_[24]:

(3) $$F=-\frac{dU}{dx}=-\frac{1}{2}\frac{dC_{r}}{dx}V^{2}$$

guarantees that all harmonics of these frequencies will be created. The signals at ω_s_ ± ω_m_ are of particular interest because they can then be re-radiated back to the drive coil and detected, allowing remote determination of the mechanical frequency, ω_m_.

**Figure 5.**
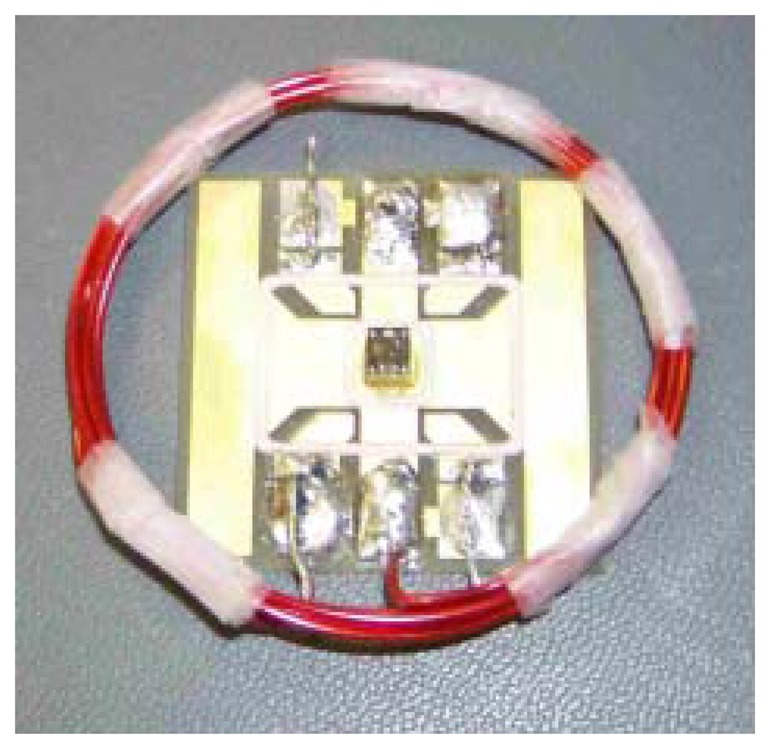
A packaged MEMS comb drive is shown in the center with die coupled inductive loop. The 4 cm square board is used simply as wirebond landings and a mechanical substrate to hold the coil. Ultimately, the coil would be printed directly on the board itself for highest reproducibility and lowest cost [22].

Figure 5 shows the packaged die where a 5-loop coil of wire resonated the die capacitance at 14.2 MHz. A second 5-loop coil of wire, stood off from the die-coupled loop by ∼1 cm, was driven by an RF synthesizer. The synthesizer output was also coupled to a diode that served to down-convert the re-radiated signal. While not the same device as shown in Figure 1, the comb drive response was shown experimentally to have a similar Q and resonant frequency driven electrically as when driven acoustically.

In order to optimize the configuration to maximize the read range and minimize the required power, a more detailed understanding of the inductive link was required, enabling a more careful design of the coils, geometry and receiver electronics. The magnetic field, B, at distance r from an N-turn coil loop of radius a carrying a current I is given by:

(4) $$B(r)=\frac{\mu_{0}IN}{2}\frac{a^{2}}{{\left(a^{2}+r^{2}\right)}^{\overset{3}{\;}/\underset{2}{\;}}}$$

If the drive current is an AC signal at the drive frequency, ω_d_, this field will induce a voltage in a second receive coil. Assuming the receive coil of N_r_ turns of area A_r_ is oriented at an angle α to the transmit coil, and is part of an LC tank circuit with an electrical quality factor Q_d_, the induced voltage is given by:

(5) $$V(r)=\omega_{d}N_{r}A_{r}Q_{d}B(r)\cos(\alpha )$$

The goal is to maximize this voltage in order to maximize the force on the comb drive capacitor. The first key constraint is that the LC resonant frequency should be close to “standardized” frequency allocations, of which the relevant ones are at 127 kHz and 13.56 MHz. Given die capacitances of about 60 pF for the silicon comb drive, frequencies between 10 MHz and 20 MHz were targeted. A simple expression for the inductance L (in mH) of an N_r_–turn coil loop of radius a_r_ (in cm) and length l (in cm) is given by:

(6) $$L=\frac{a_{r}^{2}N_{r}^{2}}{\left(22.9a_{r}+25.4l\right)}$$

where:

(7) $$A_{r}=\pi a_{r}^{2}$$

To maximize V(r) under the constraint of a fixed resonant frequency, N_r_ and A_r_ must be maximized. In practice, a 6-turn coil of 2.5 cm radius and 0.25 cm length was chosen to provide an inductance of 3.6 mH, which resonated the 58 pF of the capacitor of the comb drive at ∼11 MHz. The expected electrical quality factor, Q_d_, of the tank can be determined from the parasitic resistance, R, from:

(8) $$Q_{d}=\frac{\omega_{d}L}{R}$$

With parasitic resistances of about 16.5 Ω, the expected Q_d_ ∼15 is a little larger than the measured Q_d_ ∼12 using simple RF reflection and transmission measurements. This would be an important factor to optimize in an advanced design. Of course, the quality factor cannot get too high or it will be hard to re-radiate the 30 kHz resonant oscillations within the bandwidth of the LC tank.

The design of the read coil is another important factor in efficient delivery of power to the die. A reasonable strategy is to match the driving coil to the impedance of the driving system, which is typically 50 Ω. The magnetic field falls off as 1/r^3^ outside of the radius of the drive coil, so a large drive coil is desirable to maximize the read range. For this work a single-turn square coil, ∼15 cm on edge, was chosen and it was matched to a 50 Ω drive with a simple capacitive network.

**Figure 6.**
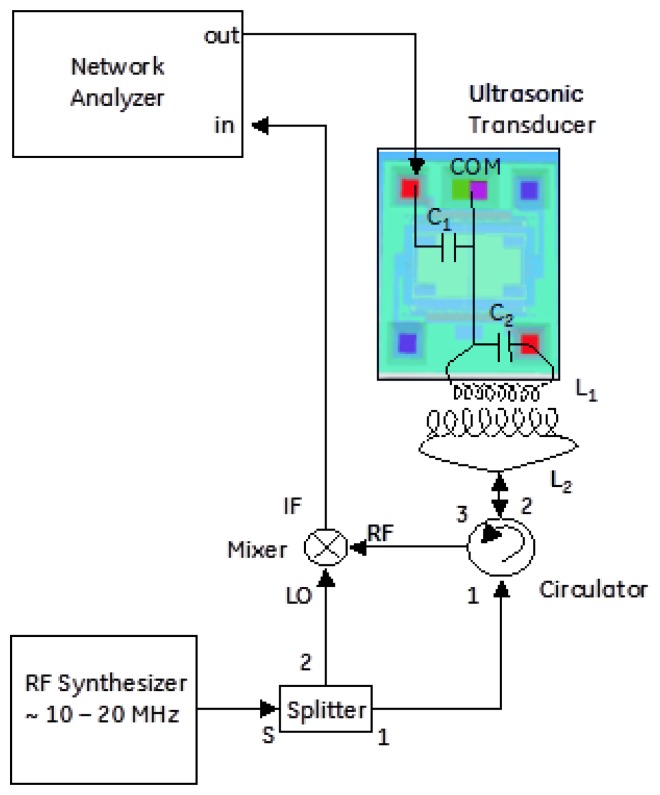
A more sophisticated and efficient receiver used a portion of the driving signal as a local oscillator to downconvert the reflected signal to “baseband” at 30 kHz in order to recover the modulation. Furthermore, a circulator was used to eliminate splitting losses between the transmitted and reflected RF signals.

**Figure 7.**
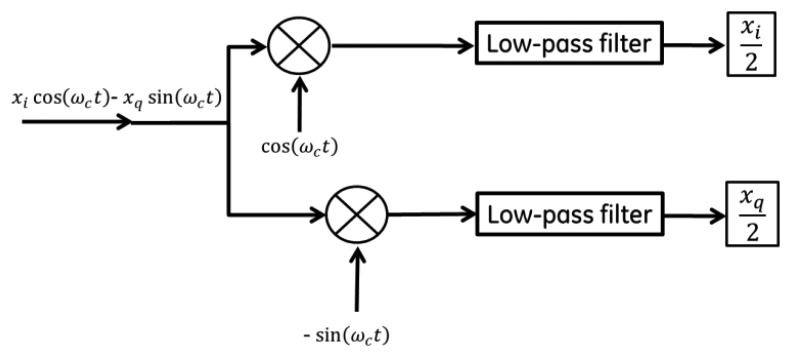
One step beyond the architecture of Figure 5 is the I/Q architecture where the relative phase between local oscillator and reflected signal is accounted for. This receiver will allow for time varying displacement between the “reader” coil and the “sensor” coil that would be found, for example, in a handheld interrogation approach. Signal processing of the I and Q legs allows the receiver to track the maximum signal amplitude, assuming the variations in relative phase are “slow” relative to the signal bandwidth.

In order to readout the signal, homodyne detection between the back-reflected signal and the original RF driving signal is performed. A very simple schematic of this setup is shown in Figure 6. In practice, the phase between the local oscillator and RF back-reflection determines the quality of the detected signal. Most measurements suggested the modulation was dominated by FM, with small residual of AM, but this would have to be studied more systematically. One step beyond the architecture of Figure 6 is the more general In-phase/Quadrature (I/Q) demodulator topology illustrated in Figure 7, where the relative phase between local oscillator and reflected signal is accounted for [25]. This would allow full recovery of amplitude and phase for a general form of modulation. This receiver architecture will also allow for time varying displacement between the “reader” coil and the “sensor” coil as would be found, for example in a handheld interrogation approach. Signal processing of the I and Q legs allows the receiver to track the maximum signal amplitude, assuming the variations in relative phase are “slow” relative to the signal bandwidth. Using this configuration, the dependence of the received signal on the relative phase between the local oscillator and the back reflection from the antenna can be eliminated.

## 4. Acoustic Drive and Inductive Read

**Figure 8.**
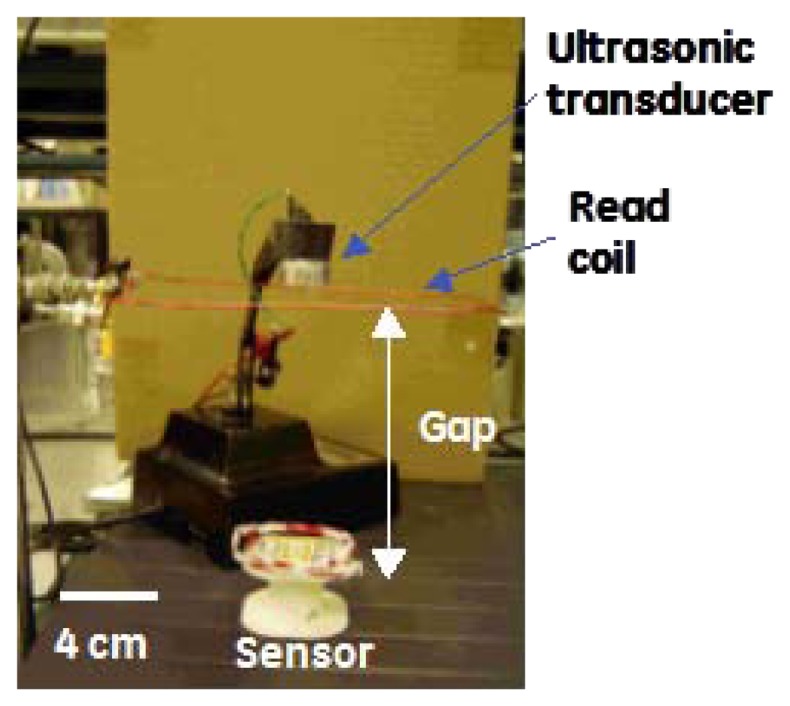
A photograph of the experimental setup for the wireless architecture shown in Figure 9.

**Figure 9.**
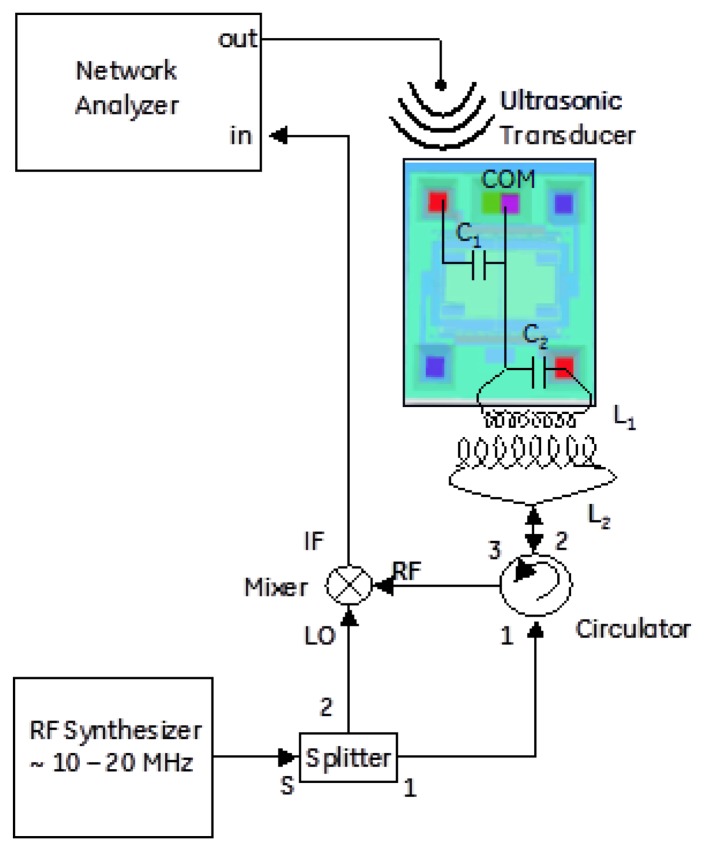
Schematic showing how the acoustic drive and inductive read techniques were combined.

Acoustic drive and inductive read were combined into a single architecture for remote standoff detection of the resonant frequency of the MEMS device. A schematic and picture of the wireless architecture is shown in Figures 8 and 9. The acoustic transducer and driving coil can be seen co-located near the middle of the picture and the sensor is shown at the bottom.

**Figure 10.**
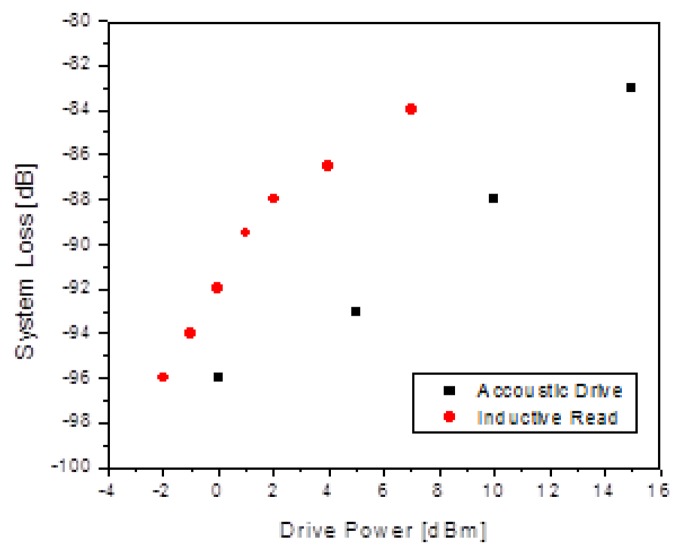
A plot of sensor received power based on acoustic and inductive drive power.

First measurements focused on the relationship of the received signal to various operating parameters as seen in Figure 10. The dependence on the acoustic drive power was in line with the expected dependence due to the “direct drive” nature of the acoustic excitation. This can be seen using Equation (1), with P_drive_ as the drive power level and p as the sound pressure level. This pressure produces a corresponding driving force, F, on the inertial mass resulting in amplitude, x_o_, of the inertial mass, with an associated motional power, P_mech_, as shown in:

(9) $$P_{\text{drive}}\propto p^{2}\propto F^{2}\propto x_{o}^{2}\propto P_{\text{mech}}.$$

However, for the inductive readout, the situation is a bit more complicated. According to Equation (3), the unmodulated RF read power, P_read_, produces a force, F, on the read capacitor proportional to this power. The amplitude of motion is proportional to F, implying a doubling of the sensitivity of the motional energy to the inductive read power level compared to the acoustic case:

(10) $$P_{\text{read}}\propto V_{\text{read}}^{2}\propto F\propto x_{0}\propto \sqrt{P_{\text{mech}}}.$$

A second important dependence was the variation of the received signal level with standoff distance, r. Based on the technical discussion above, a simple model for this dependence would look functionally like the following:

(11) $$F(r)\sim \frac{1}{{\left(a^{2}+r^{2}\right)}^{6}}\frac{1}{r^{2}}$$

**Figure 11.**
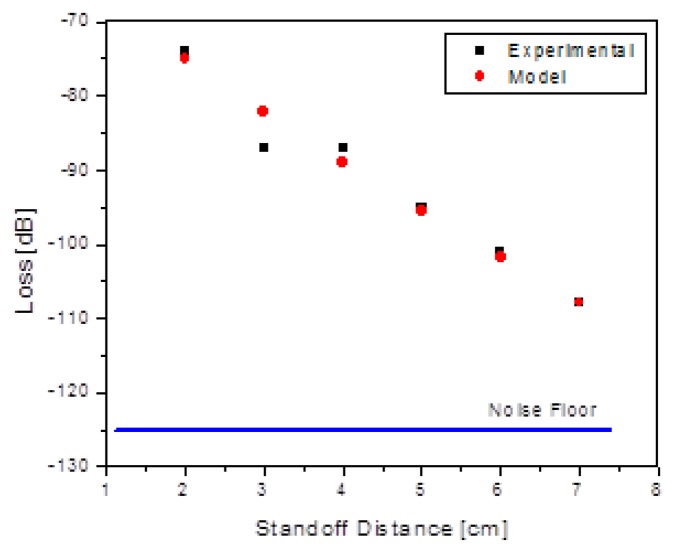
Measurements of open loop signal loss confirm the simple model of Equation (11).

The first factor in Equation (11) accounts for the fact that the voltage induced in the sensor coil in the forward direction of the inductive “link” is proportional to the magnetic field, so the power delivered to the die is proportional to the square of the magnetic field. Hence, for a 2-way link, the dependence of received signal should go as the fourth power of the B field. The second factor in Equation (11) is the square-law dependence on the acoustic drive distance. Measurements of the dependence of the received signal on standoff distance were made, and overlaid with this simple physical model. Figure 11 shows good agreement of the data with the functional form.

**Figure 12.**
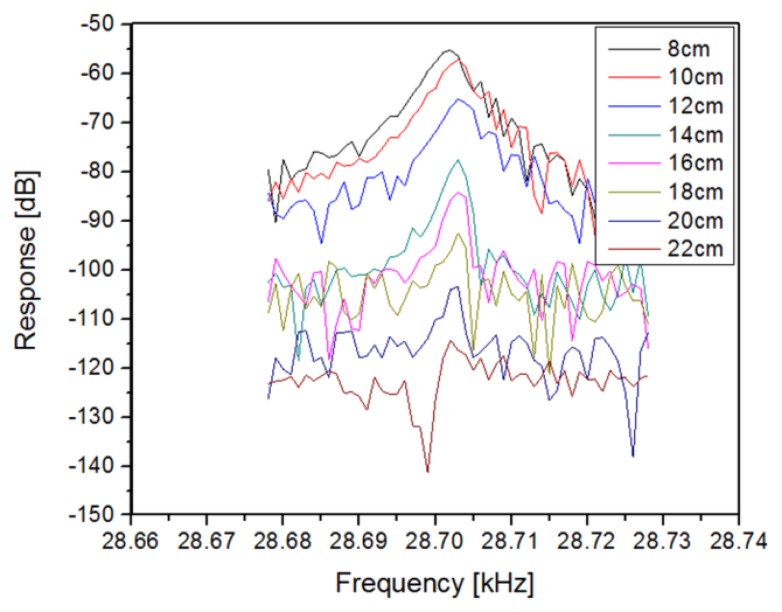
Characterization of standoff distance for the acoustic drive and inductive read using the I/Q architecture from Figure 7. The span of all measurements was 50 Hz excluding the 22 cm data which was taken at 30 Hz. For this data, the maximum of the I and Q signals was used, but in practice, they would be combined for maximum signal recovery.

With this understanding in place, the dependence of the received signal as a function of standoff distance was measured, with a maximum standoff of 22 cm achieved (Figure 12). Note that the drive levels are not constant for this data, but were set so that they did not exceed “practical” values of +15 dBm (∼30 mW). The intent was simply to find the largest standoff distance and the acoustic and inductive drive levels under this constraint. It would clearly be possible to achieve greater distances using higher drive levels for the acoustic and inductive links.

**Figure 13.**
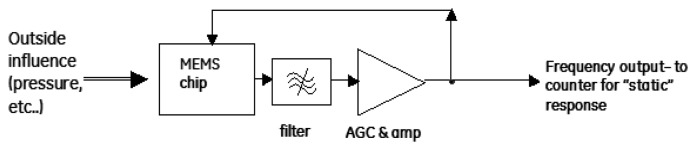
An example of a feedback loop that could be used to detect the resonance of the resonator.

One practical issue with this data is that a very expensive network analyzer was used to sweep the acoustic frequency in order to detect resonance. For low cost applications, it would be desirable to have the device “autodetect” its own resonance frequency [26]. This is typically achieved by taking the output of the detector and then applying gain and feeding it back to the drive signal as seen in Figure 13.

**Figure 14.**
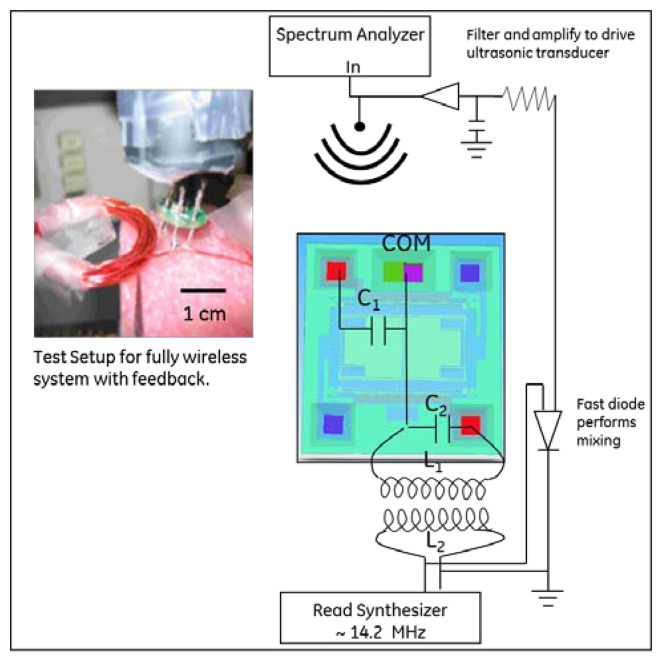
Schematic and test setup used to force the resonator into oscillation through a feedback system.

**Figure 15.**
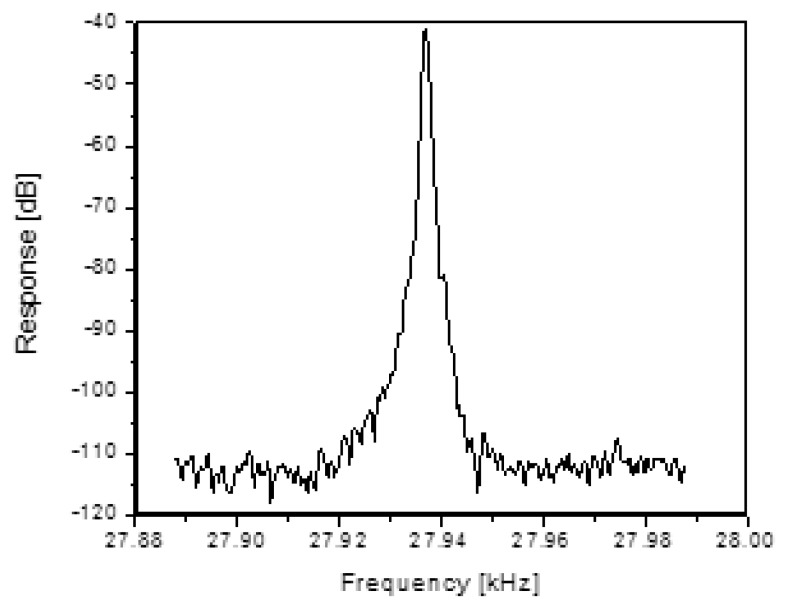
This graph shows the spectrum analyzer output when picking off the signal in some point of the feedback loop shown in Figure 14.

With enough gain, and minimal phase shift, the system can be made to oscillate on its own, from broadband noise, due to the high Q of the micromechanical resonator. A sketch of the setup used to force the system to oscillate by applying feedback with the appropriate amount of gain as shown in Figure 14. This setup eliminates the need for any expensive frequency sweeping (e.g., network analyzer) or a sophisticated method for finding the resonance frequency. After implementing this approach, the system oscillated up to a standoff distance of 15 cm, limited only by the available gain in the variable amplifier setup. Figure 15 shows an example of the sensor response when in the feedback loop shown in Figure 14. The required ∼100 dB of gain was achieved by cascading stages, and a simple multi-op-amp based design could be used in practice.

## 5. Conclusions

Using an acoustic drive and inductive readout, a micromechanical resonator was driven into resonance and wirelessly interrogated on its own from broadband noise, without the need for any junction-based electronic device (such as a diode) local to the sensor. This demonstration opens the door for the use of silicon-based high accuracy MEMS devices in high temperature wireless applications. The standoff dependence of both acoustic driving and inductive readout was determined separately and as part of the system as a whole. Oscillation from broadband noise was shown up to 15 cm away and complete wireless system with laboratory electronics up to 20 cm. Drive and readout architectures were shown that are suitable for handheld, portable applications. Further work is necessary to understand both the temperature dependence of the resonator system and the potential effects of intermediary materials between the sensor and its interrogating electronics.

## References

[1] Gould D., Sklorz A., Meiners M., Lang W., Benecke W. Passive Wireless Temperature Sensing Using RF Technology for an Automotive Application.

[2] Karthaus U., Fischer M. (2003). Fully integrated passive UHF RFID transponder IC with 16.7 um minimum RF input power. IEEE J. Solid-State Circuits.

[3] DeHennis A., Wise K. (2005). A wireless microsystem for the remote sensing of pressure, temperature, and relative humidity. J. Microelectromech. Syst..

[4] Suster M., Ko W., Young D. (2003). An optically powered wireless telemetry module for high temperature MEMS sensing and communication. J. Microelectromech. Syst..

[5] Takeuchi S., Shimoyama I. (2002). Selective drive of electrostatic actuators using remote inductive powering. Sens. Actuators A.

[6] Schimetta G., Dollinger F., Scholl G., Weigel R. Wireless Pressure and Temperature Measurement using a SAW Hybrid Sensor.

[7] Kalinin V. Passive Wireless Strain and Temperature Sensors Based on SAW Technology.

[8] Yen T.-T., Lin C.M., Zhao X., Senesky D.G., Hopcroft M.A., Pisano A.P. Characterization of Aluminum Nitride Lamb Wave Resonators Operating at 600 °C For Harsh Environment RF Applications.

[9] Takahata K., Gianchandani Y. (2008). A micromachined capacitive pressure sensor using a cavity-less structure with bulk metal/elastomer layers and its wireless telemetry applications. Sensors.

[10] Akar O., Tay A., Najafi K. (2000). A wireless batch sealed absolute capacitive pressure sensor. Sens. Actuators A.

[11] Fonseca M., English J., von Arx M., Allen M. (2002). Wireless micromachined ceramic pressure sensor for high temperature applications. J. Microelectromech. Syst..

[12] Radosavljevic G., Zivanov L., Smetana W., Maric A., Unger M., Nad L. (2009). A wireless embedded resonant pressure sensor fabricated in the standard LTCC technology. IEEE Sens..

[13] Welham C., Gardner J., Greenwood J. (1996). A laterally driven micromachined resonant pressure sensor. Sens. Actuators A.

[14] Welham C., Greenwood J., Bertioli M. (1999). A high accuracy resonant pressure sensor by fusion bonding and trench etching. Sens. Actuators A.

[15] Greenwood J., Wray T. (1993). High accuracy pressure measurement with a silicon resonant sensor. Sens. Actuators A.

[16] Greenwood J., Satchell D. (1988). Miniature silicon resonant pressure sensor. IEE Proc. D Control Theory Appl..

[17] Bartels O. The Passive Tyre-Pressure Transponder: Specifics of the Implementation. http://www.iqmobil.de.

[18] Bartels O. (2002). Apparatus for Wire-Free Transmission from Moving Parts. U.S. Patent 6,378,360.

[19] Challener W., Knobloch A., Ajgoankar M., Chamarthy P., Xia H. Subsystem Design and Validation for Optical Sensors for Monitoring Enhanced Geothermal Systems.

[20] Palit S., Challener W., Lopez J., Mandal S., Xia H. A Multi-Modality Fiber Optic Sensing Cable for Monitoring Enhanced Geothermal Systems.

[21] Challener W., Palit S., Craddock R., Knobloch A. MOEMS Pressure Sensors for Geothermal Well Monitoring.

[22] Vernooy D., Knobloch A., Ahmad F., Sexton D. Remote Excitation and Readout of a High Q Silicon Resonator.

[23] Greenwood J. (2003). Sensor. U.S. Patent 6,584,864.

[24] Koskenvuori M., Tittonen I. (2008). Towards micromechanical radio: Overtone excitations of a microresonator through non-linearities of the second and third order. J. Microelectromech. Syst..

[25] Krauss H., Bostian C., Raab F. (1980). Solid State Radio Engineering.

[26] Nguyen C., Howe R. (1999). An integrated CMOS micromechanical resonator high Q oscillator. IEEE J. Solid-State Circuits.

